# Compromised DNA Repair Promotes the Accumulation of Regulatory T Cells With an Aging-Related Phenotype and Responsiveness

**DOI:** 10.3389/fragi.2021.667193

**Published:** 2021-05-11

**Authors:** Daan K. J. Pieren, Noortje A. M. Smits, Sandra Imholz, Bhawani Nagarajah, Conny T. van Oostrom, Renata M. C. Brandt, Wilbert P. Vermeij, Martijn E. T. Dollé, Teun Guichelaar

**Affiliations:** 1 Centre for Infectious Disease Control, National Institute for Public Health and the Environment (RIVM), Bilthoven, Netherlands,; 2 Centre for Health Protection, National Institute for Public Health and the Environment (RIVM), Bilthoven, Netherlands,; 3 Department of Molecular Genetics, Erasmus MC, Rotterdam, Netherlands,; 4 Princess Máxima Center for Pediatric Oncology, Utrecht, Netherlands,; 5 Oncode Institute, Utrecht, Netherlands

**Keywords:** aging, ercc1, DNA damage, nucleotide excision repair, T cells, regulatory T cells, mTOR, rapamycin

## Abstract

Decline of immune function during aging has in part been ascribed to the accumulation of regulatory T cells (Tregs) and decreased T-cell responses with age. Aside from changes to T cells that occur over a lifetime, the impact of intracellular aging processes such as compromised DNA repair on T cells remains incompletely defined. Here we aimed to define the impact of compromised DNA repair on T-cell phenotype and responsiveness by studying T cells from mice with a deficiency in their DNA excision-repair gene *Ercc1*. These *Ercc1* mutant (*Ercc1*^−*/Δ7*^) mice show accumulation of nuclear DNA damage resulting in accelerated aging. Similarly to wild-type aged mice, *Ercc1*^−*/Δ7*^ mice accumulated Tregs with reduced CD25 and increased PD-1 expression among their naive T cells. *Ercc1-*deficiency limited the capacity of Tregs, helper T cells, and cytotoxic T cells to proliferate and upregulate CD25 in response to T-cell receptor- and IL-2-mediated stimulation. The recent demonstration that the mammalian target of rapamycin (mTOR) may impair DNA repair lead us to hypothesize that changes induced in the T-cell population by compromised DNA repair may be slowed down or reversed by blocking mTOR with rapamycin. *In vivo* dietary treatment of *Ercc1*^−*/Δ7*^ mice with rapamycin did not reduce Treg levels, but highly increased the proportion of CD25^+^ and PD-1^+^ memory Tregs instead. Our study elucidates that compromised DNA repair promotes the accumulation of Tregs with an aging-related phenotype and causes reduced T-cell responsiveness, which may be independent of mTOR activation.

## INTRODUCTION

The phenotype and functionality of T cells change during the course of aging, which contributes to aging-related pathology, increased susceptibility to infectious diseases, and reduced vaccine efficacy in the elderly (Goronzy and Weyand, 2013). The decline in T-cell function with age can in part be explained by changes to their phenotype and proliferative capacity, often referred to as T-cell exhaustion and/or T-cell senescence (Akbar and Henson, 2011). Additionally, we and others have shown that FoxP3^+^ regulatory T cells accumulate with age (Gregg et al., 2005; Nishioka et al., 2006; Sharma et al., 2006; Lages et al., 2008; Elyahu et al., 2019; Pieren et al., 2019) and it is thought that these cells impair protective immune responses by their suppressive capacity (Sharma et al., 2006; Lages et al., 2008; Garg et al., 2014). Insight into biological processes that contribute to decreased T-cell function and the accumulation of regulatory T cells (Tregs) with age is required to better understand the process of T-cell aging.

Characteristics of T-cell aging are mostly investigated on a chronological scale, i.e., changes that occur among T cells in relation to progressing time. Indeed, the exposure of antigens over a lifetime causes major alterations to the T-cell compartment (Farber et al., 2014). Aside from this standard pathway, aging is also driven by cell intrinsic processes such as the accumulation of nuclear DNA damage; a hallmark of aging and recently defined as the factor driving all other hallmarks of aging (López-Otín et al., 2013; Vermeij et al., 2016). Accumulation of DNA damage with age is a result of suboptimal DNA repair and is thought to be one of the drivers of cellular senescence (Herbig et al., 2006; Gorbunova et al., 2007; Akbar et al., 2016). This may also apply to aging of T cells, as T cells with a highly differentiated phenotype accumulate with age (Henson et al., 2009) and express higher levels of DNA damage (Lanna et al., 2014). However, to what extent characteristics of T-cell aging are explained by suboptimal DNA repair remains unclear. In this study, we defined the impact of compromised DNA repair on hallmarks of T-cell aging.

The endonuclease complex ERCC1-XPF mediates the repair of a broad variety of DNA lesions: 1) bulky helix-distorting lesions are removed by global-genome nucleotide excision repair; 2) lesions blocking transcription are removed by transcription-coupled repair; 3) DNA crosslinks are removed via interstrand crosslink repair; and 4) a subset of persisting double-strand DNA breaks are removed by the single-strand annealing pathway (Gregg et al., 2011; Marteijn et al., 2014). Mice with a deficiency in the DNA excision-repair gene *Ercc1* (*Ercc1*^−/Δ7^) have one knock-out and one truncated allele of *Ercc1* and therefore show impaired DNA repair within the four aforementioned pathways, which results in the accumulation of nuclear DNA damage (Niedernhofer et al., 2006; Vermeij et al., 2016; Robinson et al., 2018). As a consequence, *Ercc1*^−/Δ7^ mice show numerous age-related pathologies and signs of accelerated aging with a reduced average lifespan of only 20 weeks (Weeda et al., 1997; Dolle et al., 2011; Vermeij et al., 2016). *Ercc1*^−/Δ7^ mice therefore provide an aging model that enables investigation of the impact of compromised DNA repair on T-cell phenotype and responsiveness.

Another important driver of cellular aging is the activation of the mammalian target of rapamycin (mTOR) (Johnson et al., 2013). mTOR is a well-known target in anti-aging research as inhibition of mTOR by rapamycin has been widely reported to slow down the process of aging in terms of life- and healthspan (Chen et al., 2009; Harrison et al., 2009; Bitto et al., 2016). Moreover, dietary supplementation of rapamycin in an encapsulated form (eRapa) to aged mice has been shown to reduce age-related changes observed in T-cells (Hurez et al., 2015). Interestingly, activation of the mTOR kinase mTORC1 has been reported to impair DNA damage response signaling, leading to accumulation of unrepaired DNA lesions (Xie et al., 2018). Moreover, mTORC1 activation may negatively interfere with the ataxia telangiectasia mutated (ATM) checkpoint that promotes DNA repair (Shen and Houghton, 2013). We therefore hypothesized that changes to the T-cell compartment induced by compromised DNA repair may be slowed down by *in vivo* blocking of mTOR by eRapa.

In this study, we defined the impact of compromised DNA repair on T-cell phenotype and T-cell responsiveness in *Ercc1*^−/Δ7^ mice. Additionally, we assessed whether the impact of compromised DNA repair on T cells could be avoided by inhibition of mTOR by *in vivo* dietary treatment with eRapa. We present evidence suggesting that compromised DNA repair promotes the aging-related accumulation of Tregs and reduced T-cell responsiveness that we find in wild-type (WT) aged mice, which appears to be independent of mTOR activation.

## MATERIALS AND METHODS

### Mice

Young (2 months of age) and aged (22 months of age) wild type C57BL/6 female mice were purchased from Envigo (Venray, Limburg, Netherlands). Young and aged mice were maintained at the animal facilities of the Institute for Translational Vaccinology (Bilthoven, Utrecht, Netherlands). Generation and characterization of *Ercc1*^−*/Δ7*^ mice has been previously described (Weeda et al., 1997). Breeding stocks of the parental strains, i.e., Ercc1^+/−^ mice in a pure C57BL6J background and *Ercc1*^*+/*Δ*7*^ mice in a pure FVB background were generated and maintened as described (Weeda et al., 1997; Dolle et al., 2011). Genetically uniform F1-hybrid C57BL6-FVB *Ercc1*^−*/Δ7*^ mice were generated by combining both parental strains. Typical unfavorable characteristics, such as blindness in the FVB background or deafness in the C57BL6J background, do not occur in this hybrid background. Animals were housed in individual ventilated cages under specific pathogen-free conditions (20–22°C; 12 hr. light: 12 hr. dark cycle) and provided food and water *ad libitum*. Since *Ercc1*^−*/Δ7*^ mice are smaller, food was administered within the cages and water bottles with long nozzles were used from ~2 weeks of age. Wild-type F1 *Ercc1*^*+/+*^ littermates at the indicated ages were used as controls. Male and female *Ercc1*^−/Δ7^ and *Ercc1*^*+/+*^ mice were maintained at the animal facilities of the Erasmus Medical Center (Rotterdam, Zuid-Holland, Netherlands). Distribution of males and females per group; n = 3 males and n = 3 females in the group of *Ercc1*^*+/+*^ mice, n = 3 males and n = 4 females in the group of *Ercc1*^*−/Δ7*^ mice, and n = 3 males and n = 3 females in the group of *Ercc1*^−/Δ7^ mice fed with eRapa.

### Dietary Treatment With Rapamycin

Diets were based on AIN93G, using 2.3 g/kg choline chloride instead of choline bitartrate (Research Diet Services, Wijk bij Duurstede, the Netherlands). Microencapsulated rapamycin (eRapa) and empty microcapsules (Eudragit S100) were obtained from Southwestern Research Institute (San Antonio, TX, United States). 42 mg eRapa, containing 10% Rapamycin, was added per kg AIN93G food mix, resulting in a 42ppm rapamycin supplemented diet. For the control diet, 38 mg empty microcapsules were added per kg AIN93G food mix. The diets were processed into pellets which were radiated with 9 kGy (Isotron, Ede, Netherlands). Supplemented eRapa and control diets were supplied *ad libitum* to the mice at 8 weeks of age for the remainer of their life as described previously (Birkisdottir et al., 2021).

### Preparation of Single Cell Suspensions and Proliferation Labeling

Spleens of all mice were homogenized through a cell strainer to prepare single cell suspensions and red blood cells were lyzed on ice with ACK lysis buffer (0.155M NH4Cl; 10 mM KHCO3; 0.1 mM Na2EDTA, pH 7.2–7.4). Subsequently, splenocytes were resuspended in PBS to 10*10^6^ cells/mL and labeled with 0.5 μM CellTrace^™^ Violet (Invitrogen, Carlsbad, CA, United States) in PBS per milliliter of splenocyte suspension for 20 min at 37°C to track T-cell proliferation. Cells were washed in ice-cold RPMI-1640 medium (GIBCO, Thermo Fisher Scientific, Waltham, MA, United States) containing 10% fetal calf serum (FCS) (Greiner Bio-One, Kremsmünster, Austria).

### Splenocyte Culture and *In Vitro* Stimulation

To investigate T-cell proliferation and upregulation of the activation marker CD25, splenic single cell suspensions were exposed to soluble anti-CD3 (0.019 μg/ml; clone 145–2C11, eBioscience, San Diego, CA, United States) alone or in the presence of soluble anti-CD28 (0.5 μg/ml; clone PV-1, Southern Biotech, Birmingham, AL, United States) or recombinant murine IL-2 (0.1 μg/ml; eBioscience). Stimuli were prepared in RPMI-1640 medium containing 10% FCS and then added to splenocyte suspensions (4*10^5^ cells/well). Cells were cultured in 96-well U-bottom plates (CELLSTAR, Greiner Bio-One) at 37°C and 5% CO_2_ for four days.

### Immunofluorescence Labeling and Flow Cytometric Analyses

Splenic single cell suspensions were washed with PBS containing 2% FCS and labeled for 30 minutes at 4°C for a combination of cell surface markers with the following fluorescently labeled anti-mouse antibodies: anti-CD4-BUV395 (clone GK1.5), anti-CD44-V450 (clone IM7) (BD Horizon, Franklin Lakes, NJ, USA); anti-CD25-PE-Cy7 (clone PC61.5) (eBioscience); CD122-PE-Dazzle 594 (clone TM-beta1), and anti-PD-1-BV785 (clone 29F.1A12) (BioLegend, San Diego, CA, United States). Live/Dead^™^ Fixable Aqua Dead Cell Stain Kit (Invitrogen) was included in the cell surface labeling to assess cell viability. Cells were subsequently labeled intracellularly according to the FoxP3 Transcription Factor staining buffer set protocol (eBioscience) with anti-CD3zeta-FITC (clone H146–968) (Abcam, Cambridge, Cambridgeshire, United Kingdom) and anti-FoxP3-eFluor660 (clone 150D/E4) (eBioscience). Labeled cells were detected on a BD LSRFortessa X-20 (BD Biosciences, Franklin Lakes, NJ, United States). Data analysis was performed using FlowJo software (Tree Star, Ashland, OR, United States).

### Dimensionality Reduced Analyses

Dimensionality reduced analysis (viSNE) of flow cytometry data was performed in Cytobank (www.cytobank.org) (Amir et al., 2013). Cell density maps show clustering of CD4^+^ and CD4^−^ naive and memory T-cell populations that were generated from pooled flow cytometry datafiles of *Ercc1*^−*/Δ7*^ mice (n = 7) and pooled flow cytometry datafiles of the different *Ercc1*^*+/+*^ mice (n = 6). The number of cells included in the viSNE analysis were equal between *Ercc1*^-−*/Δ7*^ and *Ercc1*^*+/+*^ mice; 2.3*10^5^ naive CD4^+^ T cells, 1.4*10^5^ naive CD4^−^ T cells, 3.3*10^4^ memory CD4^+^ T cells, and 2.2*10^4^ memory CD4^−^ T cells. Clustering was based on expression of FoxP3, CD25, CD122, and PD-1. Expression of the designated cellular markers in the heatmaps was based on their ArcSinh5-transformed median expression.

### Statistics

Statistical analyses were performed using GraphPad Prism 7 software (La Jolla, CA, United States). The appropriate parametric or non-parametric tests were used based on the tested normality of distribution of the data by Kolmogrov-Smirnov test and Shapiro-Wilk test. Dependent on the number of comparisons and the normality of distribution of the data, unpaired Student *t* Test, Mann-Whitney *U* test, or Oneway ANOVA test followed by Holm-Sidak multiple comparisons test were performed as indicated in the figure legends. Data presented in bar graphs are expressed as the mean ± standard deviation (SD). For all analyses, *p* values < 0.05 were considered statistically significant.

## RESULTS

### Compromised DNA Repair Contributes to Increased Proportions of Memory T Cells

**FIGURE 1 |:**
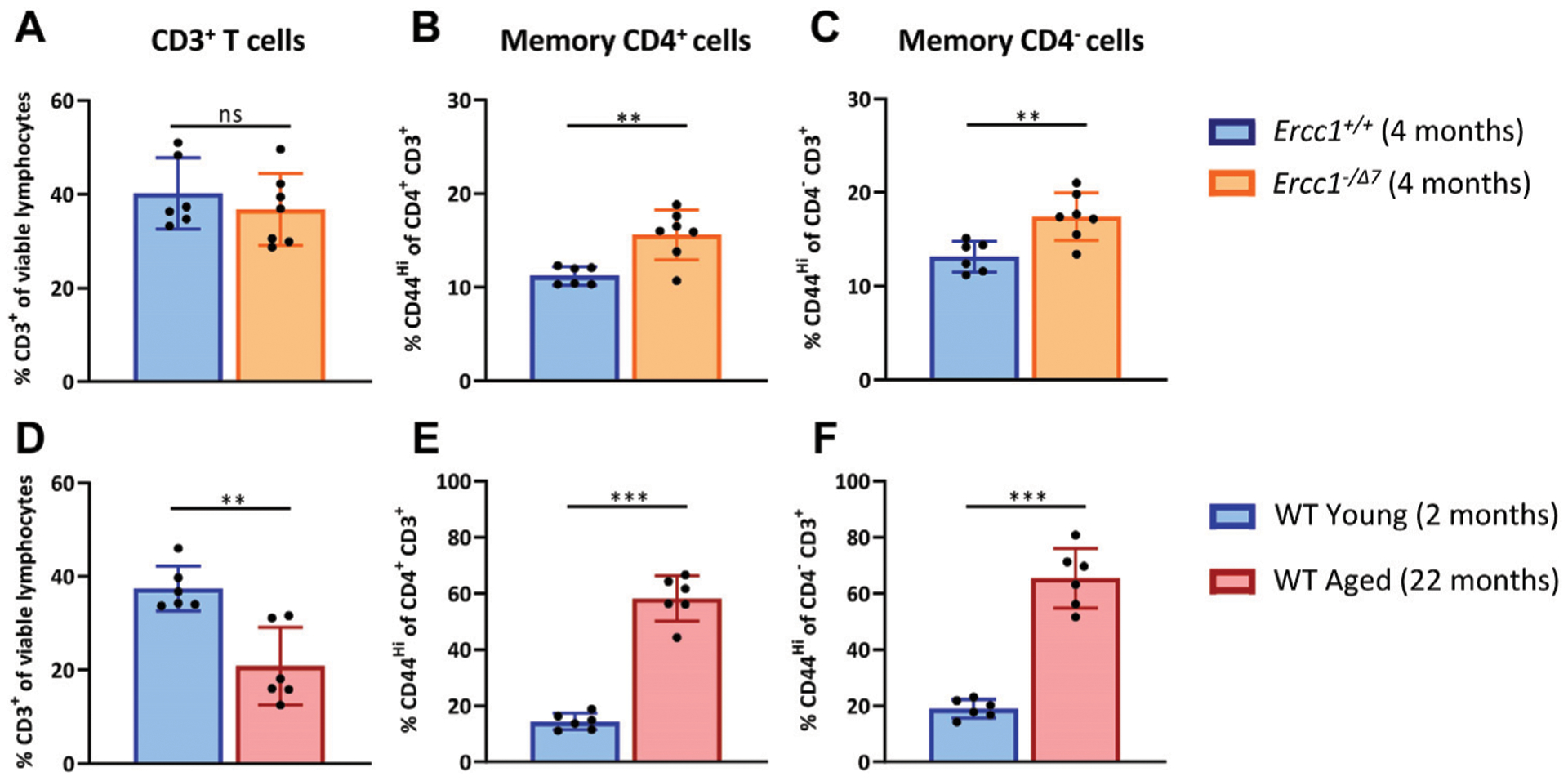
Compromised DNA repair contributes to increased proportions of memory T cells. The frequencies of **(A)** total T cells (CD3^+^ cells of viable lymphocytes), **(B)** memory CD4^+^ T cells (CD44^Hi^ of CD4^+^CD3^+^ cells), and **(C)** memory CD4^−^ T cells (CD44^Hi^ of CD4^−^CD3^+^ cells) were determined in the spleen of *Ercc1*^*+/+*^ (n = 6, 4 months of age) and *Ercc1*^*−/Δ7*^ (n = 7, 4 months of age) mice, as well as in **(D–F)** WT young (n = 6, 2 months old) and aged mice (n = 6, 22 months old). Bar graphs show mean ± SD; **p < 0.01, ns = not statistically significant for the difference between groups using unpaired Student’s *t* test, two-tailed.

With age, the total T-cell population among lymphocytes decreases, whereas the proportion of memory cells within the T cell population increases (Farber et al., 2014; Duggal et al., 2019; Pieren et al., 2019). We first assessed whether compromised DNA repair contributes to these aging-related changes. We compared the T-cell populations in spleens of accelerated aging *Ercc1*^*-*−*/Δ7*^ mice (n = 7, 4 months of age) with those in spleens of littermate control *Ercc1*^*+/+*^ mice (n = 6, 4 months of age) (Figures 1A–C). As a reference we did similar analyses in WT young (n = 6, 2 months of age) and WT aged (n = 6, 22 months of age) mice (Figures 1D–F).

*Ercc1-*deficient mice did not significantly show a decrease of CD3^+^ T-cell frequency among live spleen lymphocytes (Figure 1A) that occured in normal WT aging (Figure 1D). Subsequently, we analyzed the frequency of memory cells by expression of the memory marker CD44 among CD4^+^ T cells and CD4^−^ T cells. Of note, CD3^+^ CD4^−^ T cells can be considered CD8^+^ T cells as this subset mainly comprises CD8^+^ T cells and a very low proportion of CD4^−^ CD8^−^ cells in *Ercc1*^−*/Δ7*^ mice that we analyzed in an additional data set (3% on average) (Supplementary Figure S1), similar to previous observations in WT mice (4% on average) (Pieren et al., 2019). Although *Ercc1*^−*/Δ7*^ mice did not reach the major increase of memory CD4^+^ and CD4^−^ T cell frequencies found in WT aged mice (Figures 1E,F), the proportion of memory T cells was higher in *Ercc1*^−*/Δ7*^ mice compared to control *Ercc1*^*+/+*^ mice (Figures 1B,C). Thus, our data indicate that the rise in proportion of memory T cells among CD4^+^ and CD4^−^ T cells found in WT aged mice can in part be attributed to compromised DNA repair.

### Compromised DNA Repair Promotes Accumulation of FoxP3^+^ Tregs Within the Naive CD4^+^ T-Cell Subset

Elevated frequencies of Tregs among the CD4^+^ T-cell pool is a major hallmark of aging. Age-related differences in T-cell subsets have largely been ascribed to the shifted balance of naive T cells toward memory T cells during aging rather than an aging-related effect within these cell subsets. Indeed, Tregs in aged WT mice showed a shift toward increased numbers of memory cells in the Treg pool (Supplementary Figure S2 (Raynor et al., 2015). Although *Ercc1*^*-*−*/Δ7*^ mice showed elevated frequencies of memory cells in the overall T-cell pool (Figures 1B,C), they did not show a different memory cell frequency among Tregs compared to control *Ercc1*^*+/+*^ mice (Supplementary Figure S2). However, the impact of aging may be reflected beyond the mere shift in balance of naive/memory T cells and may be defined by changes within naive and memory T cell populations (Pieren et al., 2019). Indeed, we previously reported that naive CD4^+^ T cells of WT aged mice are enriched with FoxP3^+^ Tregs that express an aging-related phenotype characterized by increased expression of PD-1 and lower expression of CD25 (Pieren et al., 2019). Here we asked whether this major hallmark of aging in WT aged mice can be attributed to compromised DNA repair. We applied dimensionality reduction (viSNE) to form phenotypically distinct clusters based on simultaneous expression of different aging-related molecules within CD4^+^ naive (CD44^Lo^) and memory (CD44^Hi^) T cells of *Ercc1*^−*/Δ7*^ mice and *Ercc1*^*+/+*^ mice (Supplementary Figure S3, gating strategy). Cluster formation was based on the combined expression of FoxP3, CD25, CD122, and PD-1 as aging-related markers previously used to reveal aging-related clusters of T cells (Pieren et al., 2019).

**FIGURE 2 |:**
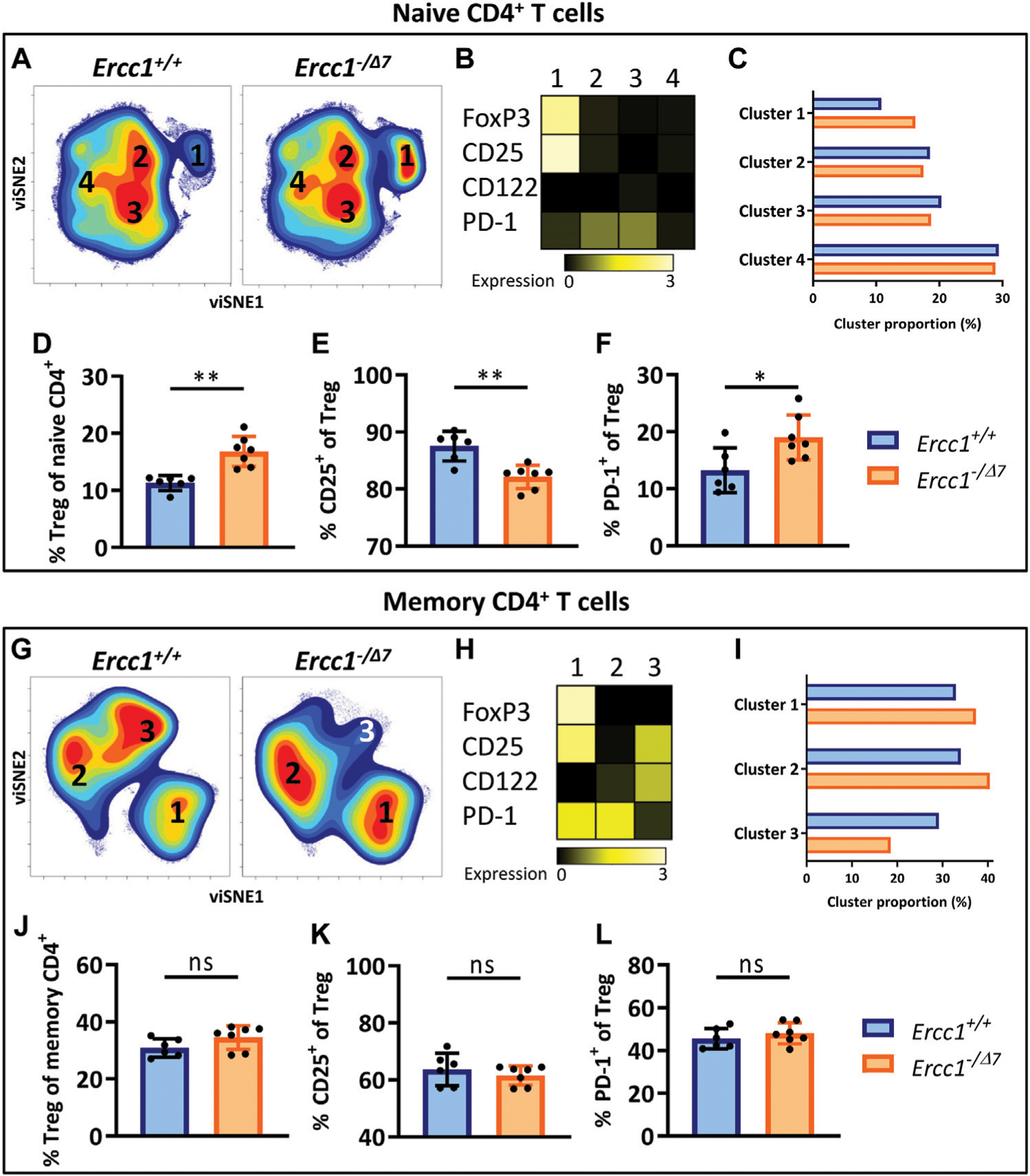
Compromised DNA repair promotes accumulation of FoxP3^+^ Tregs within the naive CD4^+^ T-cell subset. Naive (CD44^Lo^) and memory (CD44^Hi^) cells were identified within the CD4^+^ T-cell population. Cell density maps of dimensionality reduced single-cell data by viSNE show clustering within **(A)** naive CD4^+^ T cells and **(G)** memory CD4^+^ T cells of pooled flow cytometry datafiles of *Ercc1*^*+/+*^ mice (n = 6) and pooled datafiles of *Ercc1*^−*/Δ7*^ mice (n = 7). Numbers in the density maps correspond to the cluster numbers above the heat maps **(B,H)**. These heat maps depict the Arcsinh-transformed median expression of the indicated markers. Bar graphs **(C,I)** indicate the proportion of each cluster within the total viSNE for *Ercc1*^*+/+*^ (blue) and *Ercc1*^−*/Δ7*^ (orange) mice. Bar graphs **(D–F, J–L)** show the frequency of **(D)** naive and **(J)** memory FoxP3^+^ regulatory T cells in individual *Ercc1*^*+/+*^ and *Ercc1*^−*/Δ7*^ mice, as well as the frequency of **(E,K)** CD25^+^ and **(F,L)** PD-1^+^ cells within these naive and memory Treg subsets. Bar graphs show mean ± SD; **p < 0.01, ns = not statistically significant for the difference between *Ercc1*^*+/+*^ and *Ercc1*^−*/Δ7*^ mice using parametric unpaired Student’s t test or non-parametric Mann-Whitney test, two-tailed, dependent on the tested normality of distribution of the data.

viSNE analysis of T cells of *Ercc1*^*+/+*^ (n = 6 pooled) and *Ercc1*^−*/Δ7*^ mice (n = 7 pooled) showed four phenotypically distinct clusters within the naive CD4^+^ T-cell subset (Figure 2A). Cluster 1 highly expressed the Treg marker FoxP3 (Figure 2B) and the proportion of this cluster was higher in *Ercc1*^−*/Δ7*^ mice compared to *Ercc1*^*+/+*^ mice (16.2 vs. 10.8%) (Figure 2C). Accumulation of Tregs within the naive CD4^+^ T-cell subset was indeed significantly higher in individual *Ercc1*^−*/Δ7*^ mice compared to control *Ercc1*^*+/+*^ mice (Figure 2D), which closely resembled findings in WT aged mice (Supplementary Figure S4). Additionally, accumulated Tregs of *Ercc1*^−*/Δ7*^ mice comprised significantly lower CD25^+^ (Figure 2E) and higher PD-1^+^ cell frequencies (Figure 2F) and expression levels per cell (MFI) (Supplementary Figures S5 and S6) compared to *Ercc1*^*+/+*^ mice, which is similar to observations in WT aged mice (Pieren et al., 2019). Thus, compromised DNA repair appears to contribute to the accumulation of Tregs within the naive CD4^+^ T-cell compartment with an aging-related phenotype that resembles previous findings in WT aged mice (Pieren et al., 2019).

### FoxP3^+^ Tregs Within the Memory CD4^+^ T-Cell Subset in Mice With Compromised DNA Repair

WT aged mice do not accumulate Tregs within the memory CD4^+^ T-cell pool ((Pieren et al., 2019) and Supplementary Figure S4) and we assessed whether *Ercc1*^−*/Δ7*^ mice resemble these findings. viSNE analysis of memory (CD44^Hi^) CD4^+^ T cells of *Ercc1*^−*/Δ7*^ and *Ercc1*^*+/+*^ mice showed three phenotypically distinct clusters (Figure 2G) with cluster 1 indicating FoxP3-expressing Tregs (Figure 2H). Whereas the proportion of cluster 1 containing Foxp3^+^ cells was higher in *Ercc1*^−*/Δ7*^ mice compared to *Ercc1*^*+/+*^ mice (37.4 vs. 32.9%) (Figure 2I), the frequency of Foxp3^+^ Tregs within the memory CD4^+^ T-cell subset did not show a difference between individual *Ercc1*^−*/Δ7*^ and *Ercc1*^*+/+*^ mice (Figure 2J). In contrast to previous findings in WT aged mice (Pieren et al., 2019), *Ercc1*-deficiency did not show altered frequency and expression of CD25 and PD-1 on Tregs within the memory CD4^+^ T-cell subsets (Figures 2K,L; Supplementary Figures S5 and S6). These findings suggest that compromised DNA repair may not significantly contribute to phenotypical changes of Tregs within the memory CD4^+^ T-cell pool.

### The Aging-Related Phenotype of Naive Th Cells in Mice With Compromised DNA Repair

We next investigated whether compromised DNA repair contributes to phenotypical changes within naive (CD44^Lo^) helper T cells (Th cells). viSNE-clusters 2, 3, and 4 identified within the naive CD4^+^ T-cell pool do not express FoxP3 (Figures 2A,B), indicating that these cells are naive Th cells. The minimal difference in proportion of clusters 2, 3, and 4 between *Ercc1*^−^ and *Ercc1*^*+/+*^ mice (Figure 2C) indicates that *Ercc1*-deficiency did not profoundly affect the phenotype of naive Th cells based on the markers we included. Indeed, *Ercc1*^*−/Δ7*^ mice did not show the increased frequencies or expression of PD-1^+^ cells among naive Th cells (Supplementary Figure S6) that we previously found in WT aged mice (Pieren et al., 2019). Thus, these findings suggest that *Ercc1*-deficiency may not have a significant impact on expression of CD122, CD25, and PD-1 by the naive Th population and therefore might not explain the aging-related changes based on these markers within the naive Th-cell subset in WT aged mice.

### Compromised DNA Repair Promotes the Aging-Related Phenotype of Memory Th Cells

Next, we assessed the impact of compromised DNA repair on the phenotype of memory (CD44^Hi^) Th cells. These cells were represented by the FoxP3-negative clusters 2 and 3 within the memory CD4^+^ T cells in our viSNE analyses (Figures 2G,H). Cluster 2 was more abundant in *Ercc1*^−*/Δ7*^ compared to *Ercc1*^*+/+*^ mice (40.4 vs. 34.0%), which points toward more *Ercc1*^−*/Δ7*^ memory Th cells expressing PD-1 as reported previously in WT aged mice(Pieren et al., 2019). Indeed, a trend toward higher PD-1^+^ memory Th-cell frequency and higher expression of PD-1 on *Ercc1*^−*/Δ7*^ memory Th cells compared to *Ercc1*^*+/+*^ cells was found (Supplementary Figure S6). Further, we observed a lower proportion of cluster 3 (18.6 vs. 29.2%) in *Ercc1*^−*/Δ7*^ compared to *Ercc1*^*+/+*^ mice (Figure 2I), which is reflected in lower CD122 expression and CD122^+^ memory Th-cell frequency in *Ercc1*^−*/Δ7*^ mice (Supplementary Figure S5) and corresponds to earlier findings in WT aged mice (Pieren et al., 2019). Together, these data indicate that *Ercc1*-deficiency imposes an aging-related phenotype on memory Th cells that has been reported based on the expression of PD-1 and CD122.

### Aging-Related Changes in Naive and Memory Tc Cells in Mice With Compromised DNA Repair

**FIGURE 3 |:**
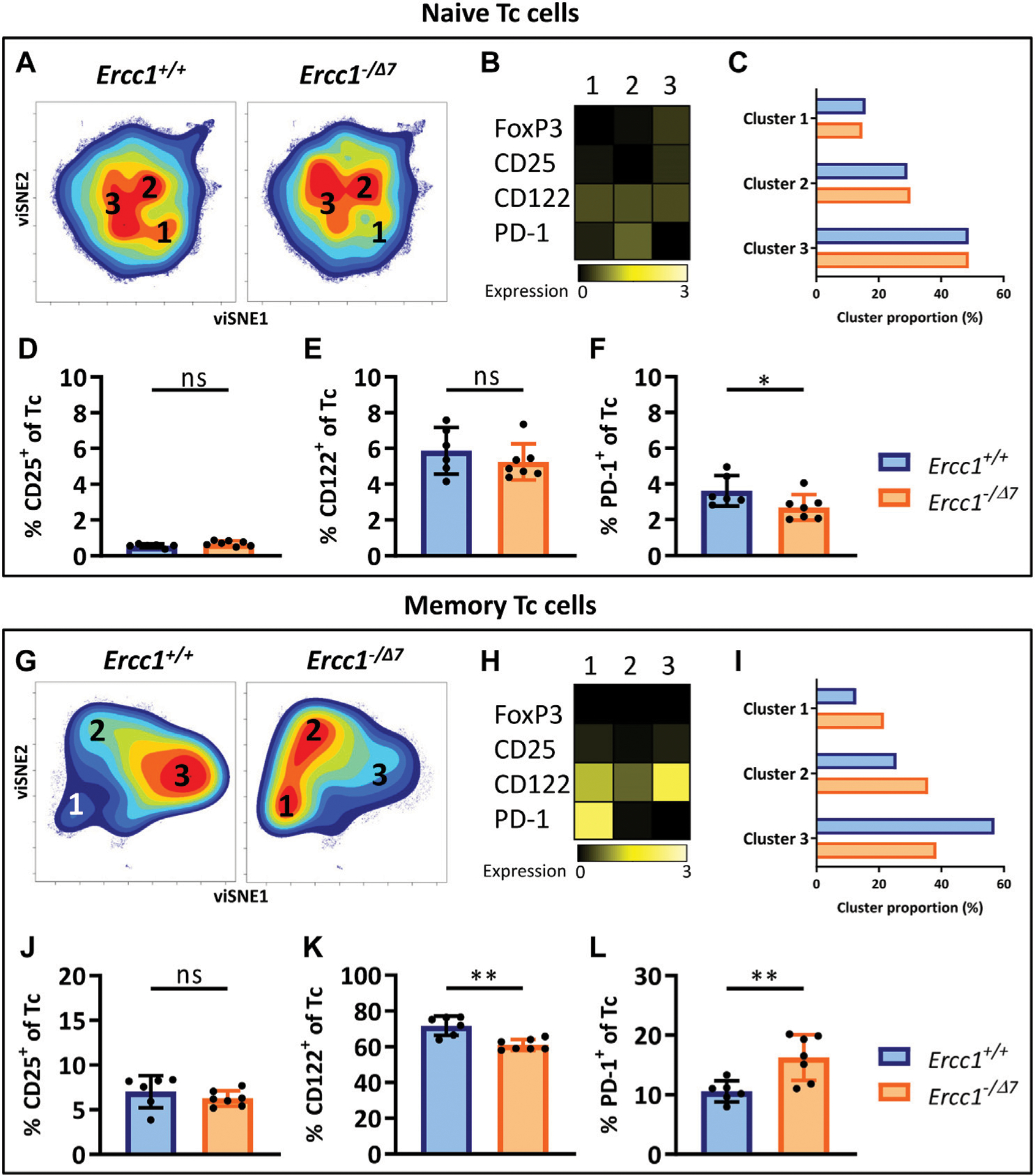
Aging-related changes in naive and memory Tc cells in mice with compromised DNA repair. Naive (CD44^Lo^) and memory (CD44^Hi^) cells were identified within the CD4^−^ T-cell population (Tc cells). Cell density maps of dimensionality reduced single-cell data by viSNE show clustering within **(A)** naive Tc cells and **(G)** memory Tc cells of pooled flow cytometry datafiles of *Ercc1*^*+/+*^ mice (n = 6) and pooled datafiles of *Ercc1*^−/Δ7^ mice (n = 7). Numbers in **(B,H)** the density maps correspond to the cluster numbers above the heat maps. These heat maps depict the Arcsinh-transformed median expression of the indicated markers. Bar graphs **(C,I)** indicate the proportion of each cluster within the total viSNE for *Ercc1*^*+/+*^ (blue) and *Ercc1*^−/Δ7^ (orange) mice. Bar graphs show the frequencies of **(D,J)** CD25^+^ **(E,K)** CD122^+^, and **(F, L)** PD-1^+^ cells among naive and memory Tc cells in individual *Ercc1*^*+/+*^ and *Ercc1*^−/Δ7^ mice. Bar graphs show mean ± SD; *p < 0.05, **p < 0.01, ns = not statistically significant for the difference between *Ercc1*^*+/+*^ and *Ercc1*^−/Δ7^ mice using parametric unpaired Student’s *t* test or non-parametric Mann-Whitney test, two-tailed, dependent on the tested normality of distribution of the data.

CD3^+^ cells that do not express CD4 can be considered CD8^+^ cytotoxic T cells (Tc cells), as this subset mainly comprises CD8^+^ T cells ((Pieren et al., 2019), Supplementary Figure S1). Clustering of naive (CD44^Lo^) Tc cells by viSNE showed relatively comparable cell-density plots, expression of phenotypical markers, and cluster proportions (Figures 3A–C) between *Ercc1*^−*/Δ7*^ and *Ercc1*^*+/+*^ mice, which suggests a low impact of *Ercc1*-deficiency on naive Tc cells. Indeed, we observed no phenotypical difference in expression and cell frequencies of naive Tc cells (Figures 3D,E; Supplementary Figures S5 and S6) apart from a slight decrease in the frequency of PD-1^+^ Tc cells of *Ercc1*^−*/Δ7*^ mice (Figure 3F). These findings are in contrast with increased PD-1^+^ cell-frequencies found among naive Tc cells of WT aged mice (Pieren et al., 2019).

Memory (CD44^Hi^) Tc cells of WT aged mice do not differ from those of WT young mice (Pieren et al., 2019). In contrast, viSNE analysis of *Ercc1*^−*/Δ7*^ memory Tc cells showed an increase in cluster 1 (21.5 vs. 12.7%) and 2 (35.7 vs. 25.6%), and a decrease in cluster 3 (38.4 vs. 56.9%) compared to *Ercc1*^*+/+*^ mice (Figures 3E–G). These differences were reflected in a reduced frequency of CD122^+^ cells and an increased frequency of PD-1^+^ cells among the memory Tc cells of *Ercc1*^−*/Δ7*^ mice compared to *Ercc1*^*+/+*^ mice (Figures 3I,J), which is also shown in the expression of these markers on a per cell basis (Supplementary Figures S5 and S6). Together, these data indicate that the impact of compromised DNA damage repair on naive and memory Tc cells may not explain findings on aging of Tc cells in WT aged mice.

### Compromised DNA Repair Promotes the Accumulation of Regulatory Cells Within the Memory Tc-Cell Subset

PD-1^+^CD122^+^ Tc cells are regulatory Tc cells (Tc reg) (Dai et al., 2010; Elizondo et al., 2019) and these cells mainly accumulate within the naive Tc-cell subet of WT aged mice (Pieren et al., 2019). In contrast, PD-1^+^CD122^+^ Tc reg cells did not accumulate within the naive Tc-cell subset of *Ercc1*^−*/Δ7*^ mice (Supplementary Figure S7), but rather within their memory Tc-cell subset. Moreover, the frequency of CD25^+^ naive and memory Tc reg cells did not differ between *Ercc1*^−*/Δ7*^ and *Ercc1*^*+/+*^ mice. Thus, compromised DNA repair does not explain findings on Tc reg cells in WT aged mice.

### eRapa Reduces Memory T Cells That Are Induced by Compromised DNA Repair

Inhibition of mTOR by rapamycin reduces aging-related T-cell changes in WT mice (Hurez et al., 2015). Moreover, the mTOR pathway is thought to impair the DNA damage response (Xie et al., 2018). We therefore hypothesized that *in vivo* dietary treatment of *Ercc1*^−*/Δ7*^ mice with eRapa may slow down changes to the T-cell compartment induced by compromised DNA repair. To this end, 8 week old *Ercc1*^*-*−*/Δ7*^ mice were fed eRapa for the remainder of their life (Birkisdottir et al., 2021) [42 ppm, reported previously as a life- and healthspan extending dose (Miller et al., 2014)].

**FIGURE 4 |:**
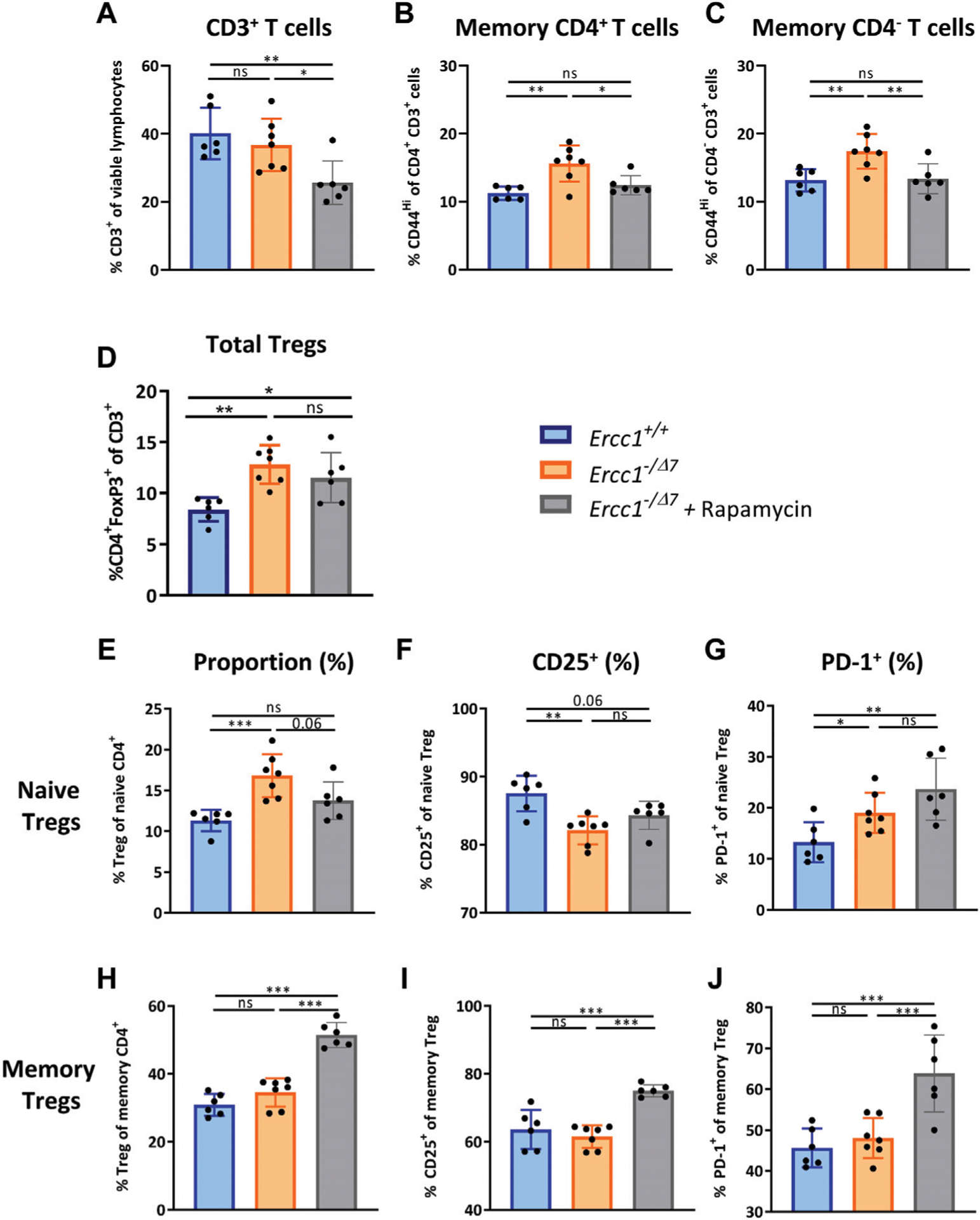
eRapa increases the proportion of Tregs within the memory CD4^+^ T-cell subset. The frequencies of **(A)** total T cells (CD3^+^ cells of viable lymphocytes) **(B)** memory CD4^+^ T cells (CD44^Hi^ of CD4^+^CD3^+^ cells) **(C)** memory CD4^−^ T cells (CD44^Hi^ of CD4^−^CD3^+^ cells), and **(D)** total Tregs (CD4^+^FoxP3^+^ of CD3^+^) were determined in the spleens of *Ercc1*^*+/+*^ (n = 6, blue bars), *Ercc*^−/Δ7^ (n = 7, orange bars), and *Ercc1*^−/Δ7^ treated with eRapa (n = 6, gray bars). The frequencies of **(E)** naive Tregs (FoxP3^+^ cells of CD44^Lo^CD4^+^CD3^+^), and **(H)** memory Tregs (FoxP3^+^ cells of CD44^Hi^CD4^+^CD3^+^), and their respective **(F,I)** CD25^+^ and **(G,J)** PD-1^+^ cell frequencies were also determined in these mice. Bar graphs show mean ± SD; *p < 0.05, **p < 0.01, ***p < 0.001, ns = not statistically significant for the difference between groups using one-way ANOVA corrected with Holm-Sidak test for multiple comparisons.

eRapa-fed *Ercc1*^−*/Δ7*^ mice showed decreased proportions of CD3^+^ T cells among live spleen-derived lymphocytes compared to control-fed *Ercc1*^*+/+*^ and *Ercc1*^−*/Δ7*^ mice (Figure 4A), consistent with previous reports in WT mice (Hurez et al., 2015). Interestingly, eRapa reduced the proportion of memory CD4^+^ and CD4^−^ T cells toward levels found in control-fed *Ercc1*^*+/+*^ mice (Figures 4B,C), suggesting that the increase of memory T cells induced by compromised DNA repair may in part be dependent on mTOR activation.

### eRapa Increases the Proportion of Memory Tregs but Does Not Reduce the Accumulation of Total Tregs

eRapa did not prevent the rise in proportion of FoxP3^+^ Tregs within the overall CD3^+^ T cell pool in *Ercc1*^−*/Δ7*^ mice (Figure 4D). However, we observed that eRapa decreased the proportion of Tregs within the naive CD4^+^ T-cell pool of *Ercc1*^−*/Δ7*^ mice and strongly increased the proportion of Tregs within the memory CD4^+^ T-cell pool of these mice (Figures 4E,H). eRapa did not prominently reverse the aging-related lower CD25^+^ and higher PD-1^+^ cell frequencies found among naive Tregs of *Ercc1*^−*/Δ7*^ mice (Figures 4F,G). In contrast, eRapa highly increased the frequency of CD25^+^ and PD-1^+^ cells among memory Tregs, which was not observed in *Ercc1*^−*/Δ7*^ mice without eRapa (Figures 4I,J). Together, these data suggest that accumulation of Tregs with an aging-related phenotype by compromised DNA repair may be independent of mTOR activation.

### Aging-Related Reduction of CD122^+^ Memory Th-Cell Frequencies can Be Restored by eRapa

One of the few observations in the Th-cell subset of *Ercc1*^−*/Δ7*^ mice that reflected findings in WT aged mice (3) was a decreased frequency of CD122^+^ cells among memory Th cells (Supplementary Figure S6). eRapa restored the proportion of CD122^+^ memory Th cells to levels found in *Ercc1*^*+/+*^ mice (Supplementary Figure S8). This finding shows that eRapa may reverse this aging-related aspect of memory Th cells and suggests that CD122 expression by memory Th cells is dependent on mTOR activation.

### Compromised DNA Repair Limits T-Cell Receptor/Interleukin-2-Mediated Treg Proliferation and Activation

Reduced proliferation and reduced upregulation of the activation marker CD25 in response to cellular stimulation are hallmarks of declined T-cell responsiveness at older age (Jiang et al., 2007; Pieren et al., 2019). To investigate the consequences of compromised DNA repair on T-cell responses, we exposed total spleen cells of *Ercc1*^*+/+*^ and *Ercc1*^−*/Δ7*^ mice, or young and aged WT mice to anti-CD3 alone to mimic stimulation of the T-cell receptor (TCR), or in combination with anti-CD28 or interleukin-2 (IL-2) as a co-stimulator. After four days, we measured T-cell responsiveness by proliferation and upregulation of the activation marker CD25. Additionally, we stimulated T cells of eRapa-fed *Ercc1*^−*/Δ7-*^ mice to assess whether eRapa would prevent consequences of compromised DNA repair on T-cell responsiveness.

**FIGURE 5 |:**
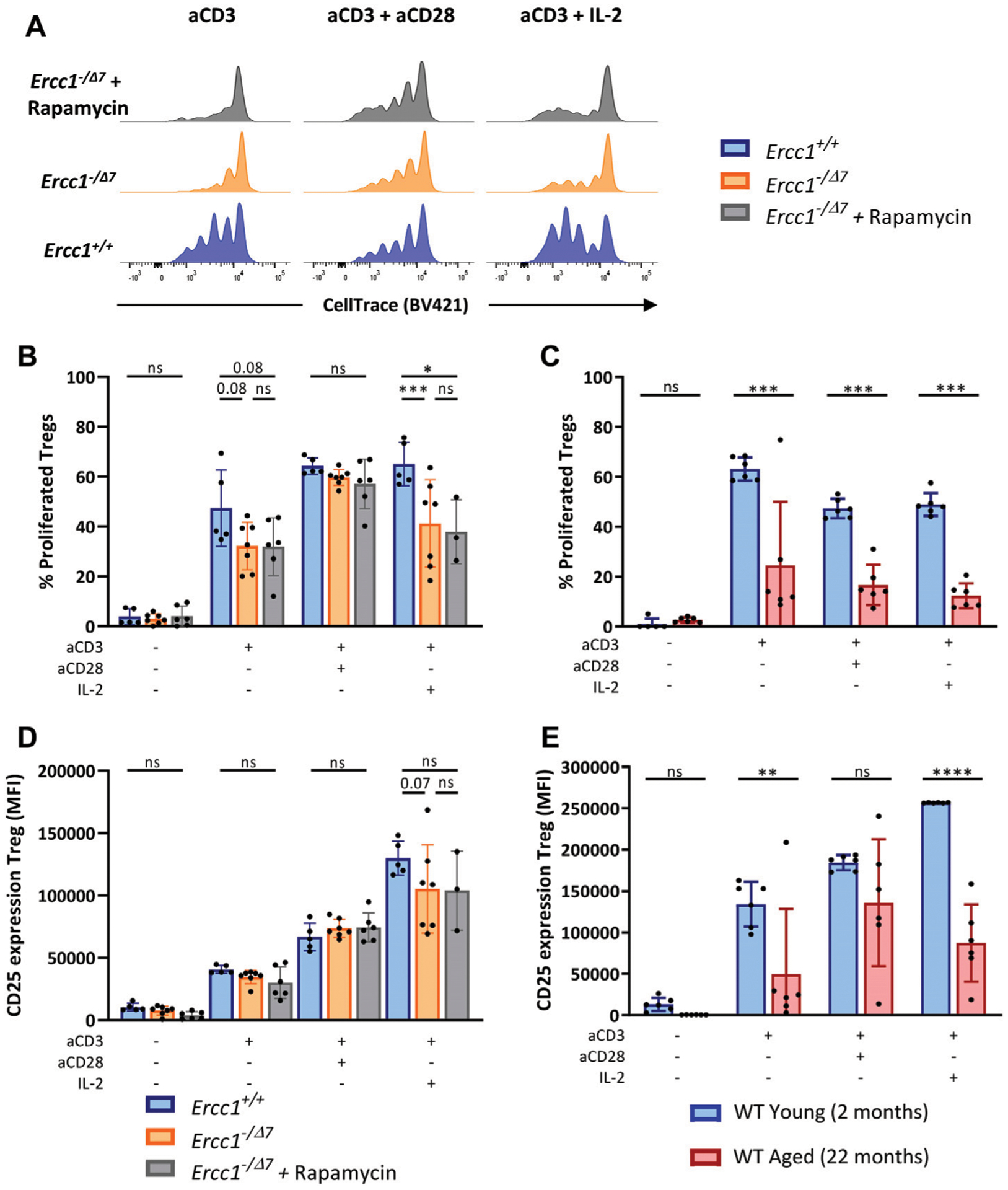
Compromised DNA repair limits T-cell receptor/Interleukin-2-mediated Treg proliferation and activation. Total splenocytes of WT young (n = 6) and aged (n = 6) mice, and *Ercc1*^*+/+*^ (n = 5), *Ercc1*^−/Δ7^ (n = 7), and *Ercc1*^−/Δ7^ mice treated with rapamycin (n = 3–6) were exposed to anti-CD3 alone or in combination with anti-CD28 or IL-2 for four days. Treg proliferation was traced by CellTrace labeling **(A)**; dilution of CellTrace label intensity indicates cellular proliferation. Bar graphs show **(B)** Treg proliferation of *Ercc1*^−/Δ7^ and *Ercc1*^−/Δ7^ mice and of **(C)** WT young and aged mice. Bar graphs show CD25 expression by Tregs of **(D)** *Ercc1*^−/Δ7^ and *Ercc1*^−/Δ7^ mice and **(E)** WT young and aged mice. Bar graphs show mean ± SD; *p < 0.05, **p < 0.01, ***p < 0.001, ns = not statistically significant for the difference between groups using parametric one-way ANOVA corrected with Holm-Sidak correction for multiple comparisons. Due to the low number of eRapa-fed *Ercc1*^−/Δ7^ mice (n = 3), statistical significance of this group was determined by non-parametric Mann-Whitney *U* Test.

We observed that compromised DNA repair in part explains diminished Treg proliferation in WT aged mice, as Tregs of *Ercc1*^−*/Δ7*^ mice showed a trend toward reduced proliferation in response to anti-CD3 alone and significantly reduced proliferation in response to anti-CD3 with IL-2 (Figures 5A,B). However, Tregs of WT aged mice also showed reduced proliferation in response to anti-CD3 combined with co-stimulation by anti-CD28 (Figure 5C), whereas CD28-mediated co-stimulation appeared to remain intact in Tregs from *Ercc1*^−*/Δ7*^ mice. eRapa did not restore the reduction of Treg proliferation observed in *Ercc1*^*-*−*/Δ7*^ mice (Figures 5A,B). Additionally, Tregs of *Ercc1* mice showed a trend toward reduced CD25 upregulation in response to anti-CD3 with IL-2, although the variation within the group of *Ercc1*^−*/Δ7*^ mice was relatively high (Figure 5D). These findings in part reflect observations in WT aged mice, as WT aged mice also showed reduced CD25 expression in response to anti-CD3 in the absence of exogenous IL-2 (Figure 5E). eRapa did not restore Treg CD25 expression levels in *Ercc1*^−*/Δ7*^ compared to *Ercc1*^*+/+*^ mice (Figure 5D). It has to be noted that the number of eRapa-fed *Ercc1*^−*/Δ7-*^ mice stimulated with anti-CD3 with IL-2 was low (n = 3) due to a limited number of splenocytes available for the different assays and these results should therefore be approached with caution. Together, our findings indicate that compromised DNA repair contributes to reduced Treg responsiveness to anti-CD3 and IL-2 observed with WT aging, which may be independent of mTOR activation.

### Compromised DNA Repair Limits T-Cell Receptor/Interleukin-2 Mediated Th- and Tc-Cell Responsiveness

Th and Tc cells of *Ercc1*^−*/Δ7*^ mice showed a trend toward reduced proliferation in response to anti-CD3 with IL-2, whereas Th and Tc cells of WT aged mice showed reduced proliferation also in response to anti-CD3 with anti-CD28 (Supplementary Figure S9). Despite the small number of mice, eRapa did not appear to restore proliferation of Th and Tc cells from *Ercc1*^−*/Δ7*^ mice in response to anti-CD3 with IL-2 (Supplementary Figure S9). Tc cells but not Th cells of *Ercc1*^−*/Δ7*^ mice showed reduced CD25 expression induced by response to anti-CD3 with IL-2, despite the individual response within each group being highly variable. In contrast, both cell types showed reduced CD25 induction in WT aged mice (Supplementary Figure S9). *In vivo* treatment of *Ercc1*^−*/Δ7*^ mice with eRapa did not affect stimulation-induced CD25 expression by their Th cells (Supplementary Figure S9). Thus, compromised DNA repair may partly explain impaired Th- and Tc-cell responses found in WT aged mice.

## DISCUSSION

Aging has a significant effect on T-cell phenotype and responsiveness, which contributes to pathology, increased susceptibility to infectious diseases, and reduced vaccine efficacy in the elderly (Goronzy and Weyand, 2013). Aside from well described T-cell aging phenomena that occur over time, the contribution of intracellular processes that drive T-cell aging such as compromised DNA repair remain incompletely defined. Here we provide novel insights into T-cell aging as we show which parts of T-cell aging can be attributed to compromised DNA repair. Moreover, we show that the majority of the changes to T cells induced by compromised DNA-repair could not be reversed by inhibition of mTOR activation.

Accumulation of Tregs during aging has been described in mice and humans (Pieren et al., 2019; Lages et al., 2008; Sharma et al., 2006; Gregg et al., 2005; Nishioka et al., 2006; Elyahu et al., 2019) and is thought to limit protective immune responses. However, the underlying cause of Treg accumulation with age remains unclear. Here we discovered that compromised DNA repair contributes to the accumulation of Tregs observed during aging. Similar to WT aged mice, Treg accumulation in *Ercc1*^−*/Δ7*^ mice was explained by an increase in the proportion of Tregs within the naive CD4^+^ T-cell subset and not within the memory CD4^+^ T-cell subset, as defined by other approaches (Supplementary Figure S2, Raynor et al., 2015). These findings indicate that propagation of memory Treg cells during aging may be a mechanism that occurs over time and complements the rise of Tregs in the naïve pool that occurs in both aging wild type mice and *Ercc1*^−*/Δ7*^ mice. Moreover, Tregs of *Ercc1*^−*/Δ7*^ mice showed an age-related phenotype characterized by increased expression of PD-1 and decreased expression of CD25, consistent with previous findings in WT mice (Elyahu et al., 2019; Pieren et al., 2019). Together, our data indicate that compromised DNA repair may be a contributing factor in promoting Treg accumulation previously observed to occur during the process of aging.

An important question that now remains is how compromised DNA repair results in the accumulation of Tregs. As possible explanation for this finding, we speculate that compromised DNA-damage repair in cells that make up the micro-environment of T cells significantly contributes to the phenotypical and functional changes found within the T cell pool with age. Senescent cells develop during the process of aging in multiple organs partly due to intracellular accumulation of DNA damage (Rodier et al., 2009; Pereira et al., 2019). These cells are known to secrete a collection of pro-inflammatory factors collectively known as the senescence-associated secretory phenotype (SASP). Accumulation of senescent cells therefore results in an inflammatory micro-environment (Rodier et al., 2009). Interestingly, it has been reported that the cytokine IL-6 is part of the pro-inflammatory SASP and promotes the accumulation of Tregs in WT aged mice (Raynor et al., 2015). Moreover, it is known that *Ercc1*^−*/Δ7*^ mice also accumulate senescent cells and show increased levels of the SASP, including higher levels of IL-6 in several organs and serum (Chen et al., 2013; Karakasilioti et al., 2013; Vermeij et al., 2016; Kim et al., 2020). Based on these and our findings, we speculate that accumulation of Tregs with age is mediated by pro-inflammatory factors like IL-6 secreted by senescent cells that result from accumulation of DNA damage over life. Accumulation of Tregs in response to IL-6 induced by DNA-damage may be an attempt by the immune system to counteract pro-inflammatory conditions that occur during aging.

Diminished T-cell proliferation is a hallmark of T-cell aging (Jiang et al., 2007; Pieren et al., 2019). Our data show that compromised DNA repair hampers TCR- and IL-2-mediated T-cell proliferation. Reduced expression of the IL-2 receptor (IL-2R) chains CD25 and CD122 may explain reduced IL-2-mediated proliferation (Nakamura et al., 1994; Liao et al., 2013). Indeed, memory Th and Tc cells of *Ercc1*^−*/Δ7*^ mice showed reduced CD122^+^ cell frequencies and expression levels. Moreover, *Ercc1*^−*/Δ7*^ Tregs and Tc cells showed reduced upregulation of CD25 expression after stimulation. Compromised DNA repair did not impact all T-cell stimulatory pathways since co-stimulation via CD28 in the presence of CD3 stimulation could trigger proliferation. This was in contrast to findings in WT aged mice as these mice show impaired proliferation in response to CD28 and CD3 stimulation. Together, our findings indicate that defects in IL-2-mediated T-cell proliferation observed with age can be attributed to compromised DNA repair. Conversely, compromised DNA repair did not hamper CD28-mediated T-cell proliferation. Therefore, impaired CD28-mediated proliferation likely develops via a mechanism other than *Ercc1*^−*/Δ7*^-mediated compromised DNA repair.

As mTORC1 negatively interferes with the ATM checkpoint that promotes DNA damage repair (Shen and Houghton, 2013), the mTOR pathway may be linked to DNA damage response signaling (Xie et al., 2018), and eRapa reduces aging-related phenotypical T-cell changes in WT mice (Hurez et al., 2015) we investigated whether rapamycin could slow down the aging-related T-cell changes imposed by compromised DNA repair. *In vivo* treatment with eRapa did not slow down the accumulation of Tregs in *Ercc1*^−*/Δ7*^ mice, suggesting that compromised DNA repair promotes the accumulation of Tregs independent of mTOR. Our findings concur with a previous study in eRapa-fed WT mice that shows no change to Treg numbers (Hurez et al., 2015). In contrast, Neff et al. report a decrease in Treg numbers after eRapa supplementation (Neff et al., 2013). These conflicting results may be explained by the difference in Treg characterization. Whereas we and others (Hurez et al., 2015) characterize Tregs by expression of the Treg master transcription factor FoxP3 (Hori et al., 2003), Neff et al. assessed Tregs as CD25^+^ cells without including FoxP3 (Neff et al., 2013). WT aging lowers the frequency and expression of CD25 on FoxP3^+^ Tregs (Nishioka et al., 2006; Pieren et al., 2019), which we also observed in *Ercc1*^−*/Δ7*^ mice. Therefore, characterization of Tregs based on CD25 only may underestimate the total amount of Tregs present.

eRapa could not prevent decreased proliferation of T cells we observed in *Ercc1*^−*/Δ7*^ mice. These findings are in contrast to previously observed findings in eRapa-fed WT mice, where eRapa improved T-cell proliferation (Hurez et al., 2015). An explanation might be that we used whole spleen cultures that allow interactions between different subsets of T cells, whereas Hurez *et al.* studied an isolated subfraction of CD4^+^ T cells. Our spleen cell cultures included eRapa-induced CD25^+^ and PD-1^+^ memory Tregs. CD25^+^ and PD-1^+^ memory Tregs of WT aged mice have been shown to comprise a Treg subset with enhanced suppressive capacity (Elyahu et al., 2019), which may suggest suppression of Th- and Tc-cell proliferation in our cultures by these eRapa-induced memory Tregs. Generation of CD25^+^ and PD-1^+^ memory Tregs by eRapa may be explained by upregulation of the memory marker CD44 on naive Tregs, since *in vivo* treatment with rapamycin can induce expression of CD44 and PD-1 FoxP3^+^ Tregs (Chaoul et al., 2015). Alternatively, memory FoxP3^−^ CD4^+^ T cells may have upregulated FoxP3 following eRapa. However, this is less likely as Foxp3^−^ effector T cells and CD4^+^CD25^−^ cells exposed *in vitro* to rapamycin do not develop into FoxP3^+^ Tregs (Battaglia et al., 2005; Charbonnier et al., 2019). Future studies on the effects of eRapa on proliferation of isolated T-cell fractions and whole-cell cultures will be important to provide full insight in the immune modulatory properties of eRapa.

Our data show that compromised DNA repair contributes to increased frequencies of memory T cells, but not to the extent found in WT aged mice. Since antigens are a major driving force behind the formation of memory T cells (Farber et al., 2014), the higher level of inflation of memory T cells found in WT aged mice is likely explained by the higher antigenic exposure over their longer lifetime. These findings indicate that compromised DNA repair partly contributes to aging-related phenotypic T-cell alterations and that aging of the T-cell pool is a diverse process that is driven by multiple factors in addition to DNA-damage. Interestingly, eRapa decreased the frequency of memory T cells found in *Ercc1*^−*/Δ7*^ mice at the benefit of a rise in the proportion of naive T cells, which is consistent with a previous report in WT mice (Hurez et al., 2015). Since higher frequencies of naive cells at young age are linked to better T-cell mediated protection, we predict that the eRapa-induced rise of the naive T cell frequency may contribute to improved T-cell mediated protection. This is supported by studies showing rapamycin improved T-cell function against pathogens (Ferrer et al., 2010) and antigen-specific T-cell responses (Araki et al., 2009).

Although our data indicate that compromised DNA damage repair contributes to aging of the immune system, there are limitations regarding our study. First, although *Ercc1*-deficient mice show compromised DNA repair in at least four repair pathways (Vermeij et al., 2016), it does not account for deficiencies in other DNA-repair pathways, such as nonhomologous end-joining or base-excision repair (Hoeijmakers, 2009). It is therefore possible that defects in DNA-repair mechanisms other than those mediated by *Ercc1-*deficiency have their own characteristic impact on aging-related T-cell changes. Second, although endogenous nuclear DNA damage accumulation in *Ercc1*^−*/Δ7*^ mice has been confirmed in liver and kidney cells (Robinson et al., 2018), it is unknown whether T cells of *Ercc1*^−*/Δ7*^ mice also accumulate DNA damage. Therefore, it remains to be established whether T-cell changes we ascribe to *Ercc1*-deficiency are due to T-cell intrinsic accumulation of DNA damage, or due to DNA damage present in the microenvironment these T cells reside in. Additionally, the inflammatory profile affecting T cells might differ between normal aging by response to antigen exposure over time, senescence-driven aging, and DNA damage-accelerated aging. Finally, we used a 42 ppm eRapa dose in our *in vivo* experiments as this dose was previously reported to further expand the health- and lifespan of WT mice compared to lower eRapa dosages (Miller et al., 2014). Compared to other studies investigating the effect of eRapa on T-cell phenotype and function in WT mice (Neff et al., 2013; Hurez et al., 2015), our dose is 3-fold higher. As different doses of rapamycin show different outcomes of lifespan in WT mice (Harrison et al., 2009; Neff et al., 2013), it is likely that changes within the T-cell population are also dose-dependent. Additionally, to what extent the 42 ppm dose of eRapa inhibits mTORC1 and mTORC2 signals remains unknown and warrants future immune studies, although recently, we did show suppression of the mTORC1 downstream effector pS6 in *Ercc1* mice by this dose of eRapa (Birkisdottir et al., 2021).

Our study uses a limited set of markers to identify T-cell subsets that may be affected. We focused on some basic aging-related T-cell subsets we and others previously identified to be subject to aging. However, the complex immune system expresses a plethora of other aging-related markers and subsets of cells that may be affected by *Ercc1*-deficiency. Thus, our data warrant future studies to unravel the impact of deficiencies of DNA-repair mechanisms on the many other aging-related molecules and T-cell subsets as well as other leukocyte subsets. For instance, markers such as CD62L that identify different memory subsets, could shed a light on central memory, effector memory, or virtual memory T-cells as well as Treg subsets. In addition, although the CD3^+^CD4^−^ population is a useful proxy for the analyses CD8^+^ T cells (without labeling CD8) that is not impacted by aging in our studies, the work we present here does not fully exclude that also minor populations of CD4^−^CD8^−^ or CD4^+^CD8^+^ T cells may be affected by *Ercc1*-deficiency. Lastly, the *Ercc1* model does not allow a fair comparison of absolute T-cell subset numbers between *Ercc1*^−*/Δ7*^ and *Ercc1*^*+/+*^ mice, as the body and organ weights of *Ercc1*^−*/Δ7*^ mice are significantly lower (Dolle et al., 2011). Smaller sizes of the spleen and the thymus in *Ercc1*^−*/Δ7*^ mice may potentially influence the output, homeostasis, and differentiation of T cells. However, the size of spleen and thymus relative to total body weight is comparable between *Ercc1*^*-*−*/Δ7*^ and *Ercc1*^*+/+*^ mice and thymic involution rates appear similar between these genotypes (Dolle et al., 2011), which may suggest minimal influence of smaller organ size on T-cell subsets.

Collectively, this study reveals a novel and pivotal role for compromised DNA repair in promoting accumulation of Tregs with an aging-related phenotype. Although smaller mTOR-mediated effects by eRapa may be missed by the relatively small group sizes in our study, compromised DNA repair appears to impose accumulation of naive Tregs through a mechanism that may be independent of mTOR activation. Our study indicates that preventing DNA-damage over the course of life may help to prevent accumulation of immunosuppressive Tregs hampering protective immunity at old age. Moreover, our study warrants further studies of biological processes that may underlie aging-related immune defects in order to better understand the process of aging.

## Supplementary Material

Supplemental Material

## Data Availability

The raw data supporting the conclusions of this article will be made available by the authors, without undue reservation.
