# Supplementary material for: Compromised DNA Repair Promotes the Accumulation of Regulatory T Cells With an Aging-Related Phenotype and Responsiveness
Source: Front Aging. 2021 May 11;2:667193. doi: 10.3389/fragi.2021.667193 (PMC9037984; doi:10.3389/fragi.2021.667193)
Supplement: Supplementary file 1 [file DataSheet1.docx]

***Supplementary Figures***

**
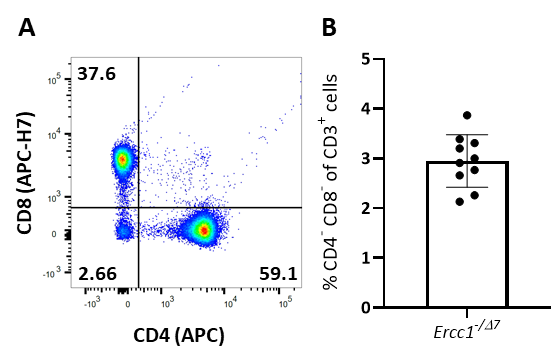
**

**Supplementary Figure 1. Gating and proportion of CD3^+^CD4^-^CD8^-^ cells in *Ercc1^-/Δ7^* mice.** Representative FACS plot (**A**) shows expression of CD8 and CD4 within the live CD3^+^ T cells of *Ercc1^-/Δ7^* mice. Numbers indicate the percentage of events in each gate as part of all events in the FACS plot. Bar graph (**B**) shows the frequency of CD4^-^CD8^-^ cells within the live CD3^+^ T-cell subset that was assessed in the spleen of *Ercc1^-/Δ7^* mice (n=10) by flow cytometry. Bar graph shows the mean ± SD.

**
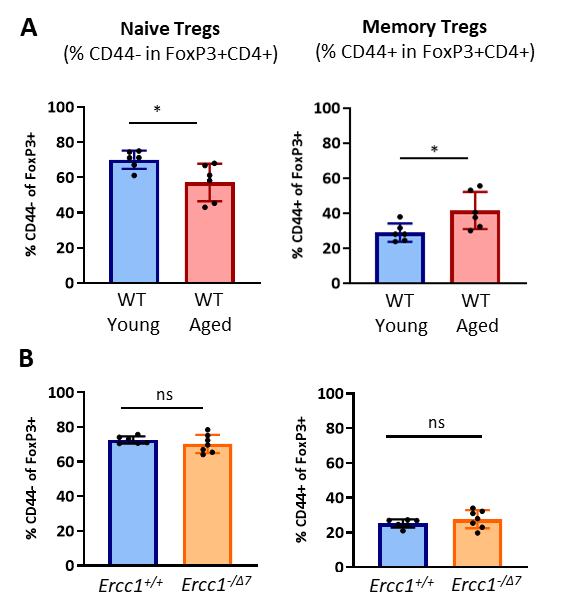
**

**Supplementary Figure 2. Frequencies of CD44^-^ naive and CD44^+^ memory cells among FoxP3^+^CD4^+^ T cells.** The frequencies of naive Tregs (CD44^-^ cells in FoxP3^+^CD4^+^ T cells) and memory Tregs (CD44^+^ cells in FoxP3^+^CD4^+^ T cells) in (**A**) WT young (n=6, 2 months old) and aged mice (n=6, 22 months old) and (**B**) *Ercc1^+/+^* (n=6, 4 months of age) and *Ercc1^-/Δ7^* (n=7, 4 months of age) mice. Bar graphs show mean ± SD; **p* < 0.05, ns = not statistically significant for the difference between groups using Mann-Whitney test.


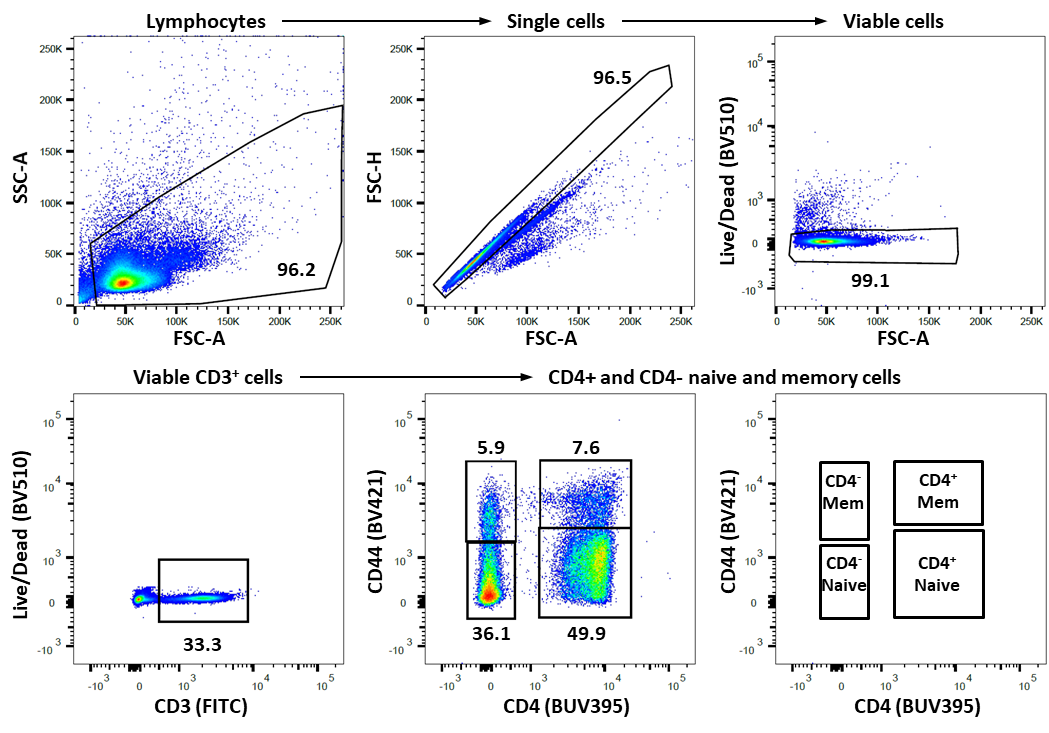


**Supplementary Figure 3. Gating strategy towards naive and memory CD4+ and CD4- T cells that were analyzed by viSNE.** Representative FACS plots show the gating from spleen lymphocytes to single cells, viable cells, and viable CD3^+^ cells. These CD3^+^ T cells were further subdivided into four T-cell subsets: naive CD4^+^ T cells (CD44^Lo^CD4^+^CD3^+^), memory CD4^+^ T cells (CD44^Hi^CD4^+^CD3^+^), naive CD4^-^ T cells (Tc cells) (CD44^Lo^CD4^-^CD3^+^), memory CD4^-^ T cells (Tc cells) (CD44^Hi^CD4^-^CD3^+^). Numbers indicate the percentage of events in each gate as part of all events in the FACS plot. The gated naive and memory cell subsets were subsequently analyzed by dimensionality reduction (viSNE).

**
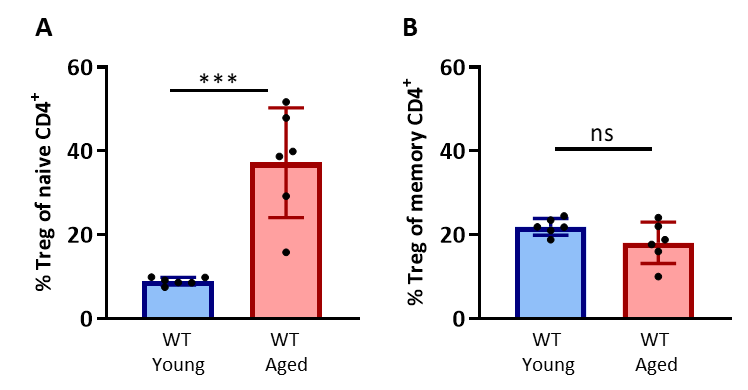
**

**Supplementary Figure 4. FoxP3^+^ Tregs accumulate within the naive CD4^+^ T cell subset of wild-type aged mice.** The frequencies of (**A**) naive Tregs (FoxP3^+^ of CD44^Lo^CD4^+^CD3^+^ cells) and (**B**) memory Tregs (FoxP3^+^ of CD44^Hi^CD4^+^CD3^+^ cells) were determined in the spleens of WT young (n=6, 2 months old) and aged mice (n=6, 22 months old). Bar graphs show mean ± SD; ***p* < 0.01, ns = not statistically significant for the difference between groups using Mann-Whitney test, two-tailed.


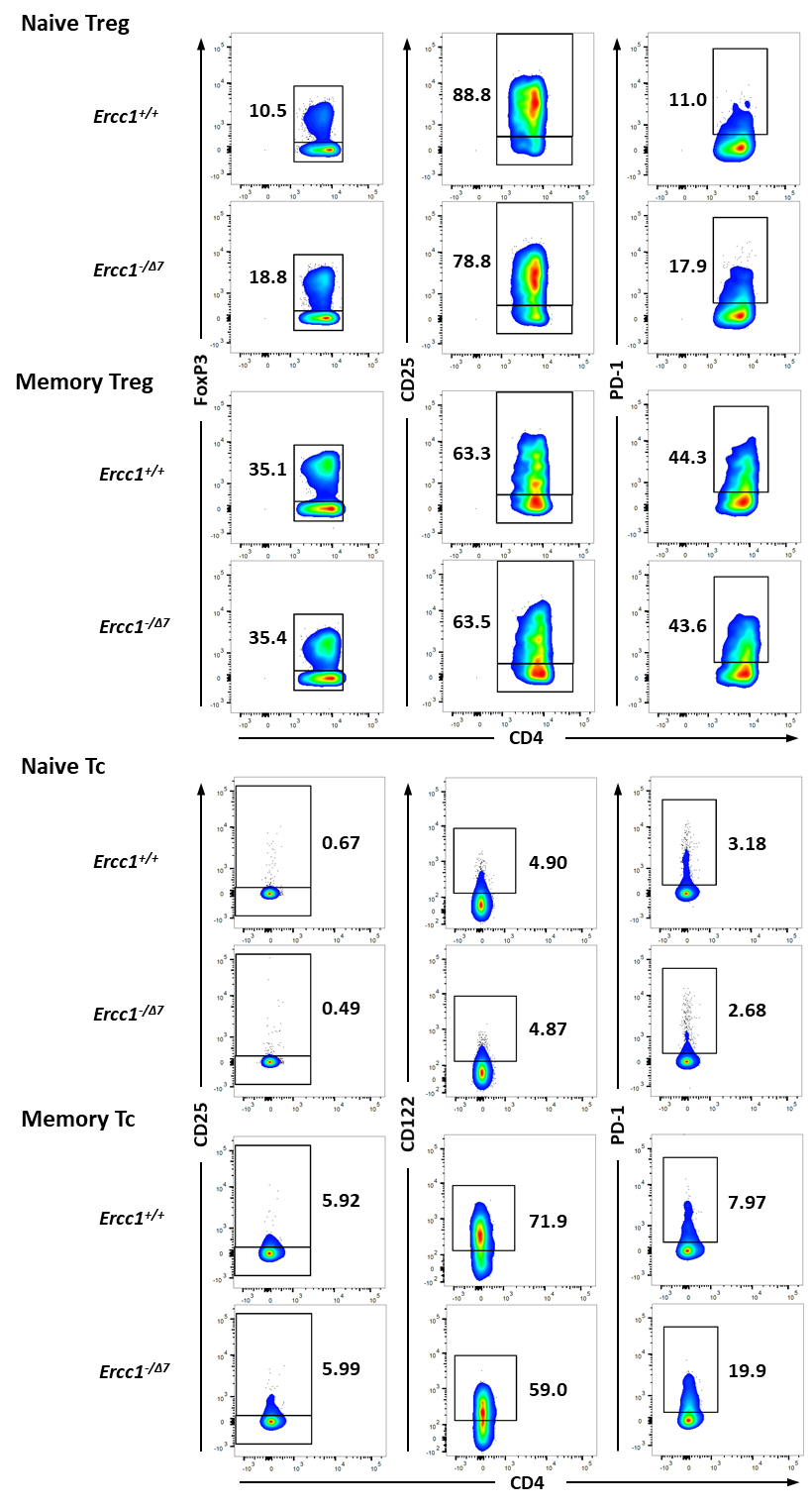


**Supplementary Figure 5. Gating of CD25, CD122, and PD-1 within naive and memory T-cell subsets.**

Representative FACS plots show gating of FoxP3, CD25, CD122, and PD-1 amongst naive Treg cells (FoxP3^+^ cells of CD44^Lo^CD4^+^CD3^+^), memory Treg cells (FoxP3^+^ cells of CD44^Hi^CD4^+^CD3^+^), naive Tc cells (CD44^Lo^CD4^-^ of CD3^+^), and memory Tc cells (CD44^Hi^CD4^-^ of CD3^+^) of *Ercc1^+/+^* and *Ercc1^-/Δ7^* mice. Numbers indicate the percentage of events in each gate as part of all events in the FACS plot.


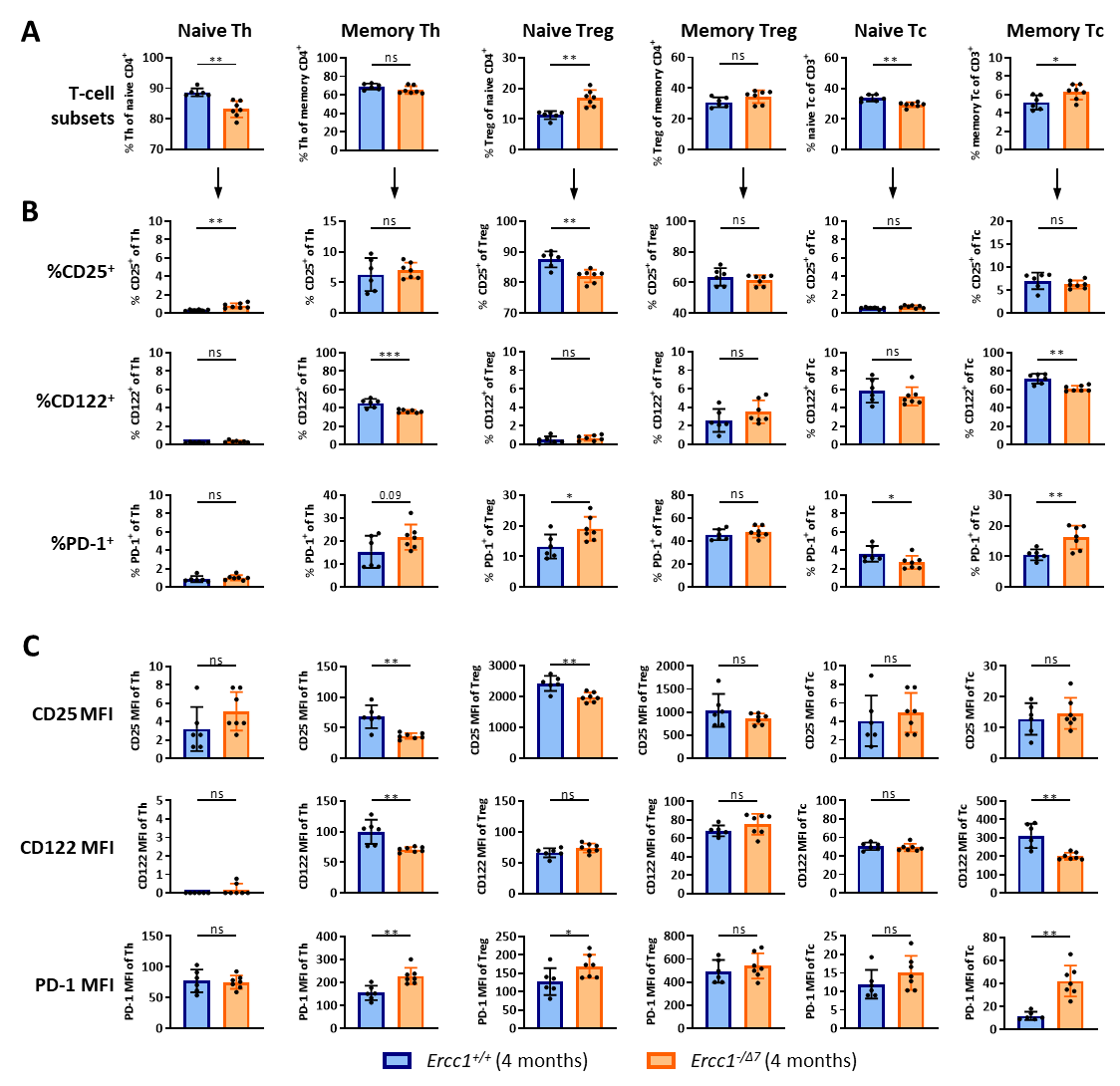


**Supplementary Figure 6. CD25, CD122, and PD-1 cell frequencies and expression within naive and memory T-cell subsets.** Bar graphs (**A**) indicate the frequencies of the following T-cell subsets: naive Th cells (FoxP3^-^ cells of CD44^Lo^CD4^+^CD3^+^), memory Th cells (FoxP3^-^ cells of CD44^Hi^CD4^+^CD3^+^), naive Treg cells (FoxP3^+^ cells of CD44^Lo^CD4^+^CD3^+^), memory Treg cells (FoxP3^+^ cells of CD44^Hi^CD4^+^CD3^+^), naive Tc cells (CD44^Lo^CD4^-^ of CD3^+^), and memory Tc cells (CD44^Hi^CD4^-^ of CD3^+^). Within each of these T-cell subsets (**B**) the frequencies of CD25^+^, CD122^+^, and PD-1^+^ cells were determined, as well as (**C**) the median expression intensity (MFI) of CD25, CD122, and PD-1. Bar graphs show mean ± SD; **p* < 0.05, ***p* < 0.01, ns = not statistically significant for the difference between groups using Mann-Whitney test, two-tailed.

**
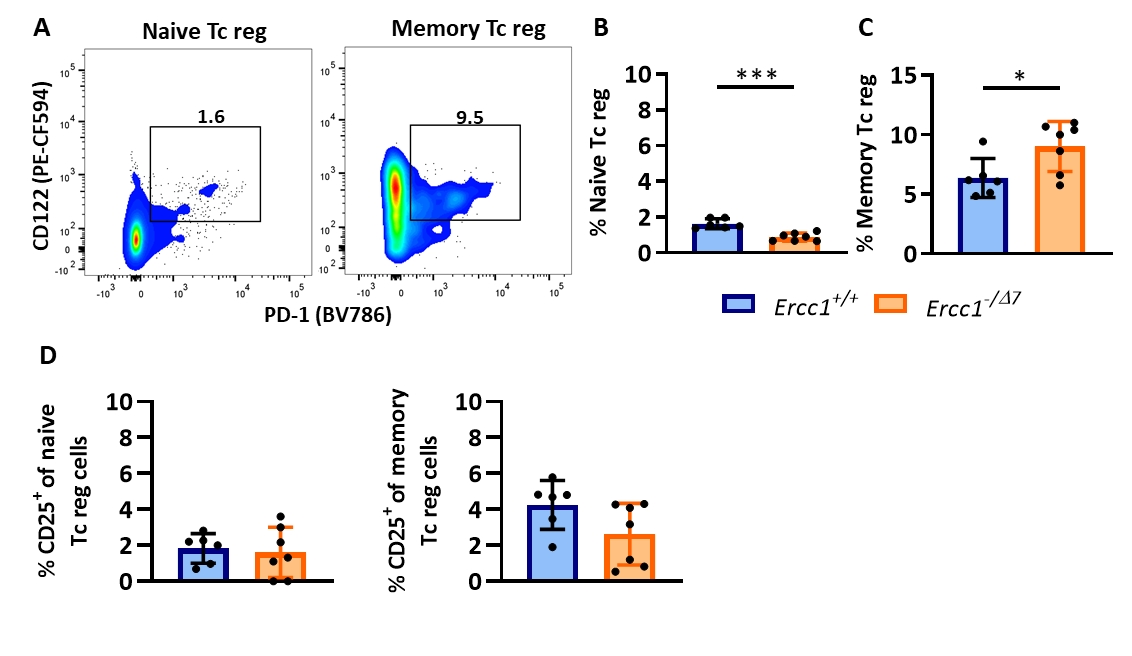
**

**Supplementary Figure 7. Tc reg cells accumulate within the memory Tc-cell subset of *Ercc1*-deficient mice.** Representative FACS plots (**A**) show the gating of naive and memory Tc reg cells based on the expression of CD122 and PD-1. The frequencies of (**B**) Treg cells among naive Tc cells (PD-1^+^ CD122^+^ of CD44^Lo^CD4^-^CD3^+^ cells) and (**C**) Treg cells among memory Tc cells (PD-1^+^ CD122^+^ of CD44^Hi^CD4^-^CD3^+^ cells) were determined in the spleen of *Ercc1^+/+^* (n=6, 4 months of age) and *Ercc1^-/Δ7^* (n=7, 4 months of age) mice, as well as (**D**) the frequency of CD25^+^ cells within these subsets. Bar graphs show mean ± SD; **p* < 0.05, ***p* < 0.01 for the difference between groups using Mann-Whitney test, two-tailed.


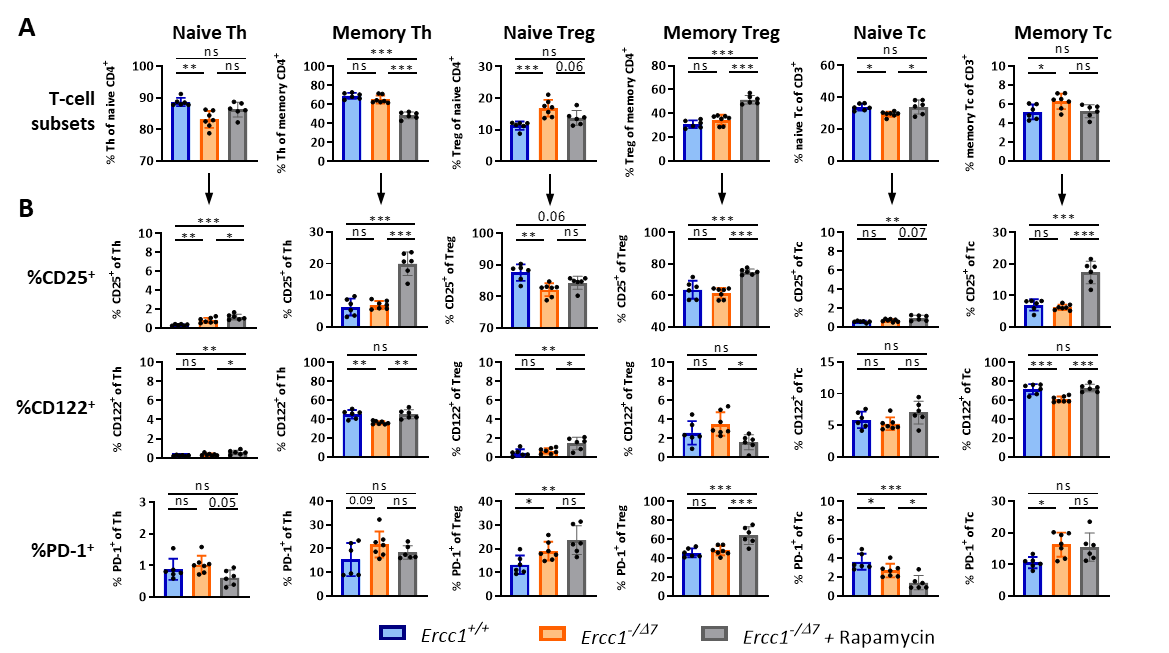


**Supplementary Figure 8. The effect of eRapa on CD25^+^, CD122^+^, and PD-1^+^ cell frequencies within naive and memory T-cell subsets.** Bar graphs (**A**) indicate the frequencies of the indicated naive and memory T-cell subsets. Within each of these T-cell subsets the frequencies of CD25^+^, CD122^+^, and PD-1^+^ cells were determined (**B**) in the spleen of *Ercc1^+/+^* (n=6, blue bars), *Ercc1^-/Δ7^* (n=7, orange bars), and *Ercc1^-/Δ7^* treated with eRapa (n=6, grey bars). Bar graphs show mean ± SD; **p* < 0.05, ***p* < 0.01, ns = not statistically significant for the difference between groups using Mann-Whitney test, two-tailed.

**
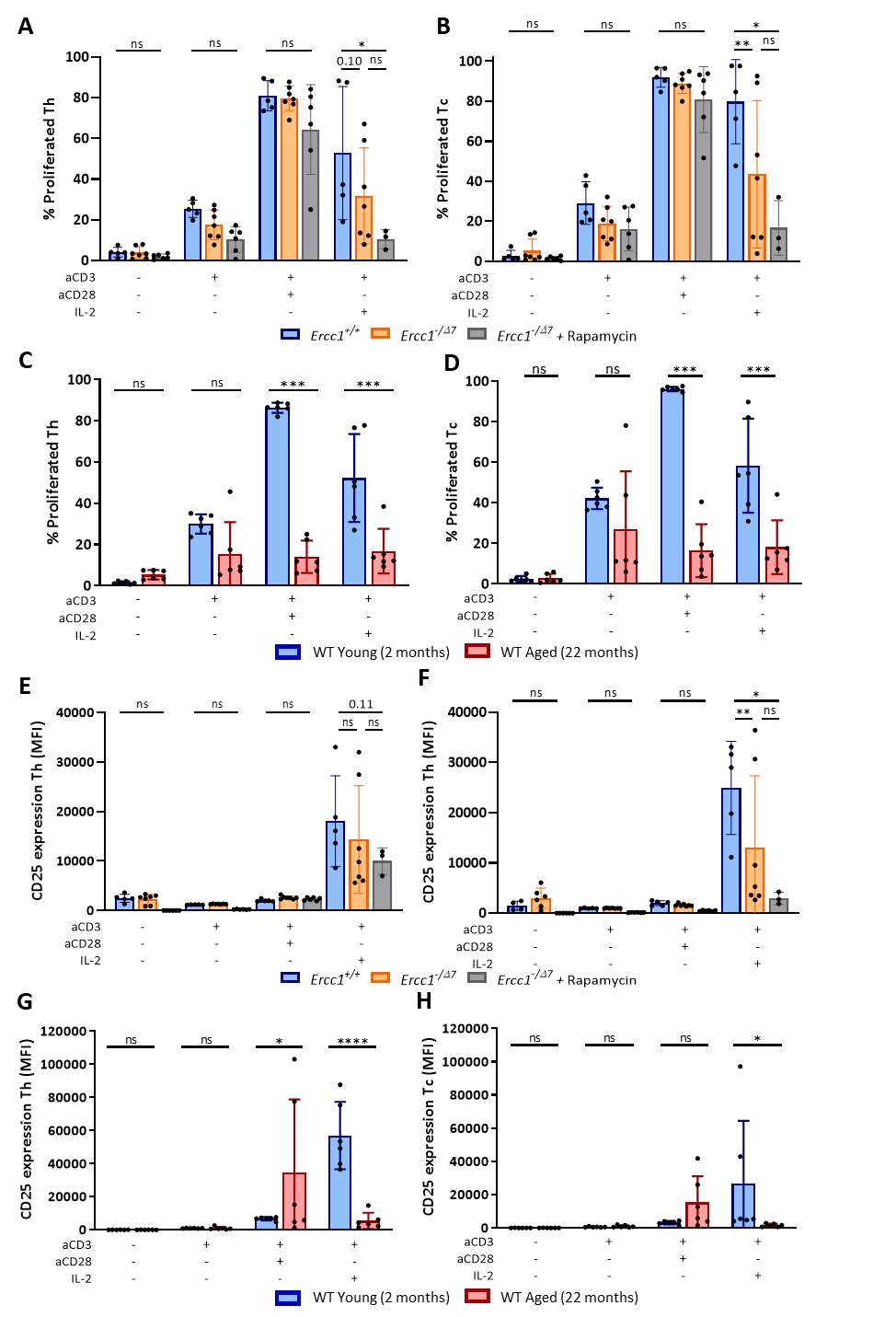
**

**Supplementary Figure 9. Defective DNA repair limits TCR/IL-2 mediated T-cell responsiveness.** Total splenocytes of WT young (n=6) and aged (n=6) mice, and *Ercc1^+/+^* (n=5), *Ercc1^-/Δ7^* (n=7), and *Ercc1^-/Δ7^* mice treated with rapamycin (n=3-6) were exposed to anti-CD3 alone or in combination with anti-CD28 or IL-2 for four days. Bar graphs show Th- and Tc-cell proliferation of (**A,B**) *Ercc1^-/Δ7^* and *Ercc1^-/Δ7^* mice and of (**C,D**) WT young and aged mice. Bar graphs show CD25 expression by Th and Tc cells of (**E,F**) *Ercc1^-/Δ7^* and *Ercc1^-/Δ7^* mice and (**G,H**) WT young and aged mice**.** Bar graphs show mean ± SD; **p* < 0.05, ***p* < 0.01, ****p* <0.001, ns = not statistically significant for the difference between groups using two-way ANOVA corrected with Sidak correction for multiple comparisons.
